# Role of *Tulipa gesneriana TEOSINTE BRANCHED1* (*TgTB1*) in the control of axillary bud outgrowth in bulbs

**DOI:** 10.1007/s00497-017-0316-z

**Published:** 2017-12-07

**Authors:** Natalia M. Moreno-Pachon, Marie-Chantal Mutimawurugo, Eveline Heynen, Lidiya Sergeeva, Anne Benders, Ikram Blilou, Henk W. M. Hilhorst, Richard G. H. Immink

**Affiliations:** 1 Physiology of Flower Bulbs, Laboratory of Plant Physiology, Wageningen University and Research, Wageningen, The Netherlands; 2 Department of Crop Science, College of Agriculture, Animal Science and Veterinary Medicine, University of Rwanda, Musanze, Rwanda; 3 Laboratory of Plant Physiology, Wageningen University and Research, Wageningen, The Netherlands; 4 Department of Plant Developmental Biology, Wageningen University and Research, Wageningen, The Netherlands; 5 Wageningen Seed Laboratory (WSL), Laboratory of Plant Physiology, Wageningen University and Research, Wageningen, The Netherlands

**Keywords:** Axillary bud, Dormancy, Apical dominance, *TgTB1*

## Abstract

**Key message:**

Tulip vegetative reproduction.

**Abstract:**

Tulips reproduce asexually by the outgrowth of their axillary meristems located in the axil of each bulb scale. The number of axillary meristems in one bulb is low, and not all of them grow out during the yearly growth cycle of the bulb. Since the degree of axillary bud outgrowth in tulip determines the success of their vegetative propagation, this study aimed at understanding the mechanism controlling the differential axillary bud activity. We used a combined physiological and “bottom-up” molecular approach to shed light on this process and found that first two inner located buds do not seem to experience dormancy during the growth cycle, while mid-located buds enter dormancy by the end of the growing season. Dormancy was assessed by weight increase and *TgTB1* expression levels, a conserved TCP transcription factor and well-known master integrator of environmental and endogenous signals influencing axillary meristem outgrowth in plants. We showed that *TgTB1* expression in tulip bulbs can be modulated by sucrose, cytokinin and strigolactone, just as it has been reported for other species. However, the limited growth of mid-located buds, even when their *TgTB1* expression is downregulated, points at other factors, probably physical, inhibiting their growth. We conclude that the time of axillary bud initiation determines the degree of dormancy and the sink strength of the bud. Thus, development, apical dominance, sink strength, hormonal cross-talk, expression of *TgTB1* and other possibly physical but unidentified players, all converge to determine the growth capacity of tulip axillary buds.

**Electronic supplementary material:**

The online version of this article (10.1007/s00497-017-0316-z) contains supplementary material, which is available to authorized users.

## Introduction

The outgrowth of axillary meristems determines the branching pattern of a plant as well as the success of vegetative reproduction for several species (Schmitz and Theres 2005). Axillary meristems arise during post-embryonic shoot development at leaf axils. Axillary meristems give rise to axillary buds, which can enter a growth arrest period after forming a few leaf primordia (Schmitz and Theres 2005). In general, after the arrest period, axillary buds sprout into lateral branches which will repeat the formation of the structures developed in the apex of the plant (Bennett and Leyser 2006). In this way, new branches are formed, but also plantlets in numerous vegetative propagated species, including various bulbous plants.

The occurrence of axillary bud outgrowth depends on the developmental stage; physiological condition of the bud (e.g. sink strength); physiological condition of the plant (e.g. apical dominance); and environmental conditions (e.g. temperature, photoperiod, light quality and nutritional availability) (Bennett and Leyser 2006; Bihmidine et al. 2013; Diaz-Riquelme et al. 2009; Horvath et al. 2003). Axillary bud outgrowth has been studied for long time in relation to apical dominance. Apical dominance is the control of the apex of the plant over the outgrowth of the axillary buds (Cline 1994). Axillary buds repressed by apical dominance are also called para-dormant, and they resume growth upon decapitation of the apex (Rinne et al. 2016). Evidence from apical dominance studies indicates that the auxin produced in the apex of the plant (Cline 1996, 2000; Cline et al. 2001; Domagalska and Leyser 2011) and the high sugar demand of the apex (Mason et al. 2014; Barbier et al. 2015) and hence, a kind of starvation of the axillary buds, are the initial inhibitory effectors in axillary bud outgrowth.

Recently, it has been suggested that sucrose is the first trigger to modulate the hormonal networks that control bud outgrowth (Barbier et al. 2015). Auxin seems to interact with the hormone strigolactone to inhibit bud growth (Bennett and Leyser 2006; Brewer et al. 2009; Liang et al. 2010), while cytokinins directly activate outgrowth (Dun et al. 2012; Shimizu-Sato and Mori 2001). Additionally, two other classical and antagonistic acting hormones, gibberellin and abscisic acid, involved in seed dormancy release and dormancy induction maintenance, respectively, also seem to play a role in the control of axillary bud development and branching (Elfving et al. 2011; Jiang et al. 2009; Reddy et al. 2013; Yao and Finlayson 2015; González-Grandío et al. 2017).

In the complex network of hormone and sugar signals controlling branching, a *TCP* transcription factor known as *TEOSINTE BRANCHED1* (*TB1*) in monocots (Doebley et al. 1997) and *BRANCHED1* (*BRC1*) in eudicots (Aguilar-Martínez et al. 2007) has been identified as master integrator (Rameau et al. 2015). A recent study, however, suggested that expression of high *BRC1* levels is not strictly needed for bud outgrowth inhibition in Arabidopsis under all environmental conditions (Seale et al. 2017). Nevertheless, *brc1* mutants in Arabidopsis present a highly branching phenotype due to the constitutive growth of the axillary buds, once the buds are set (Aguilar-Martínez et al. 2007), indicating an important axillary meristem repressing role for BRC1. Furthermore, the expression of *TB1/BRC1* genes is tightly correlated with bud activation potential in a wide range of dicot and monocot species (Manassero Nora et al. 2013). In maize, the *TB1* locus is thought to contribute to the domestication from its ancestor teosinte (Doebley et al. 1997). *TB1* overexpression in rice resulted in a reduced branching phenotype, while its mutant exhibited excessive branching (Takeda et al. 2003).

Tulip is a monocot bulbous species in which branching is of high importance because the bulb renews annually through the outgrowth of its axillary buds. Tulip bulbs are equivalent to a compacted plant whose stem shortened into a basal plate that bears modified concentric leaves (bulb scales); one central apical bud (the shoot apical meristem; SAM) and one axillary bud per bulb scale (Le Nard and de Hertogh 1993; Leeggangers et al. 2013). Note that the outer bulb scale dries out at the end of the growing season and turns into a so-called tunica, which will protect the bulb from dehydration during storage time. As a consequence, its axillary bud seems to be located on the outside of the mother bulb. The SAM of bulbs can be found in either vegetative or reproductive state, depending on the size of the bulb (De Hertogh et al. 1983), the environmental conditions and internal signals. Mature flowering-sized tulip bulbs hold on average five bulb scales and six axillary buds named from inner to outer A, B, C, D, E and H, respectively. The outermost bud is always referred to as “H-bud”. This is the bud formed from the axillary meristem in the axil of the tunica, and its name comes from the Dutch word “huid” which refers to skin (tunica) (De Hertogh et al. 1983).

Tulip bulbs need a prolonged period of winter cold to guarantee successful sprouting of the apical bud and outgrowth of the axillary buds once the temperature rises (Rebers et al. 1994). In that sense, tulip buds resemble tree apical buds. But contrary to most species, axillary bud outgrowth in tulip does not involve shoot elongation but rather “bulbing”. During this process, carbon is translocated from source organs into the scales of the axillary buds and stored into reserve compounds, hence increasing the biomass of the buds. Nevertheless, an exception to this phenomenon occurs in the H-bud, in which sprouting and anthesis take place in spring concurrent with the apical bud of the mother bulb (De Hertogh and Le Nard 1993a).

It has been reported that axillary bud activity and growth in tulip bulbs are never completely halted; it diminishes during cold and is promoted in spring (Rees 1968). Once the axillary buds grow out and develop the surrounding tunica, by the end of the summer, they receive the name of daughter bulbs. By then, the energy sources in the scales of the mother bulb have been consumed, the mother bulb senesced, and the daughter bulbs are found in “clumps” attached by the vestigial basal plate of the senesced mother bulb. For the commercial growth of tulips, this moment represents the end of the growing season, and at that moment the “clumps” are lifted from the ground, the daughter bulbs are detached, cleaned, sorted by size and stored at warm conditions (~ 20 °C) until planting time in autumn (De Hertogh et al. 1983; De Hertogh and Le Nard 1993b; Kamenetsky 2012).

In principle, every mature tulip bulb has the capacity to produce the same number of progeny bulbs as its number of axillary buds, which is on average six (Le Nard and de Hertogh 1993). However, by the end of the yearly growth cycle, only two to three daughter bulbs reach a size similar to the mother bulb and thus have the capacity to flower in the next growing cycle (Rees 1966; De Hertogh and Le Nard 1993a; Le Nard and de Hertogh 1993). Since tulip bulbs rely on axillary bud outgrowth to reproduce asexually, but this mechanism does not distribute equally the resources to all the buds, it is of great interest to understand the bases of this vegetative propagation mechanism. In this study, we aimed at identifying the factors controlling the differential axillary bud outgrowth in tulip bulbs by combining a physiological and targeted molecular approach.

## Results

### Tulip axillary bud growth in the yearly bulb growth cycle

**Fig. 1:**
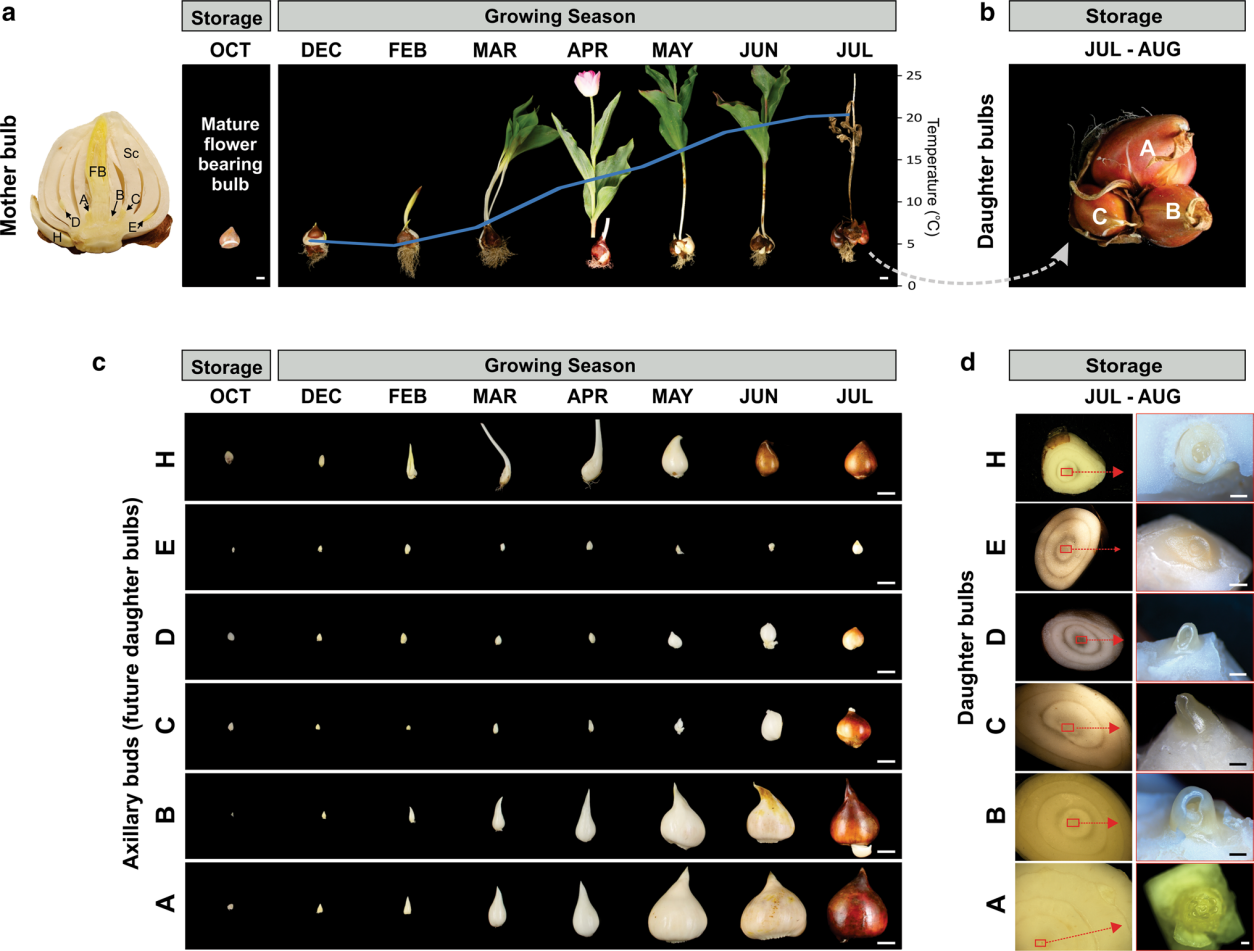
Morphological characterization of axillary bud outgrowth in a tulip bulb. **a** Structure of a flowering mother bulb prior to planting (storage period; cultivar Dynasty), and its growth development in several time points of the growing season. *A, B, C, D, E* and *H*: axillary buds located and named from inner to outer position in between the bulb scales. FB: floral bud. Sc: scales. Blue line indicates the average of the day and night temperature in the ground at 13 cm depth. Scale bar: 2 cm. **b** Clump of daughter bulbs at the end of the growing season (lifting time). *A*, *B* and *C*: daughter bulbs resulting from the outgrowth of the “*A*”, “*B*” and “*C*” axillary buds. The *D*, *E* and *H* buds are not visible from the top. **c** Outgrowth of *A*, *B*, *C*, *D*, *E* and *H* axillary buds which were dissected from the mother bulb at different time points of the growing season. The E buds dried out in 60% of the analysed bulbs. Scale bar: 2 cm. **d** Cross section of each daughter bulb at lifting time (July). The number of scales can be distinguished in the pictures, note that *A* and *B* daughter bulbs made more scales than the rest of the bulbs. Red square indicates the position of the apical meristem. Right pictures dissected apical meristem of each bulb, note that *A* and *B* buds have a floral apical bud; *H* had a floral bud in 50% of the analysed buds; and *C*, *D*, and *E* buds remained as vegetative buds. Scale bar: 1.5 mm

The natural growth cycle of tulip bulbs was monitored (Fig. 1a, b) with special emphasis on the growth of the axillary buds (Fig. 1c, d). The planted bulbs, of size 9–11 cm in perimeter, had on average five bulb scales and a tunica, six axillary buds (one axillary bud per bulb scale) and an apical shoot bearing a floral primordium (hence also called floral bud) (Fig. 1a). The bulbs were dug out at different time points during the growth season, and growth of their axillary buds was measured (Fig. 1c).

As stated by other researchers (De Hertogh and Le Nard 1993b; Le Nard and de Hertogh 1993; Rees 1968), we also found that not all axillary buds had the same growth capacity, neither the same growing behaviour (Fig. 1a–c). The outermost axillary bud (H) resembled very much an apical bud in the sense that both experienced shoot elongation, sprouting and bulb renewal (Fig. 1a). But contrary to the description of Rees (Rees 1968), which indicated that all axillary buds continue to grow during winter, although at a low rate, we did not detect growth in mid-located (C, D and E) buds during that period (Fig. 1c, from December till February, and Online resource OSM1). Once the apical bud of the mother bulb sprouted and its leaves unfolded and turned dark green (Fig. 1a), a boost in growth of A, B and H axillary buds was observed (Fig. 1c, March). Nevertheless, the growth of the mid-located buds resumed mildly, resembling a mild dormant state. By the end of the growing season (July), all axillary buds made a tunica (moment often referred to as summer dormancy), A, B and about half of the H daughter bulbs formed a reproductive apical meristem (Fig. 1d), and their A and B buds (hence, grand-daughter buds) arose later during the storage period.

The halt in growth of the mid-located buds (C, D and E buds in this study) during winter (December till February) and slow growth in early spring (March till May) suggested a differential control of axillary bud outgrowth in tulip buds, which might be determined by different levels of bud dormancy. The term dormancy as a synonym to temporary growth arrest has been redefined several times through the years mainly because one definition does not apply to all species. The concept of dormancy in geophytes is still controversial since growth of, for example, the apical bud of tulip bulbs is not arrested after its initiation (Okubo 2012). For practical reasons, we refer in this text to dormancy as the lack of sustained growth. The *TB1/BRC1* TCP transcription factor gene has been used in several dicot and monocot species as a marker to assess bud dormancy (Nicolas and Cubas 2016). To have an extra parameter to assess dormancy in tulip bulbs, we then isolated the putative *TB1/BRC1* transcript in *Tulipa gesneriana* and tested its role as a bud dormancy marker.

### Identification and gene expression of a tulip *TB1*-like TCP transcription factor

**Fig. 2:**
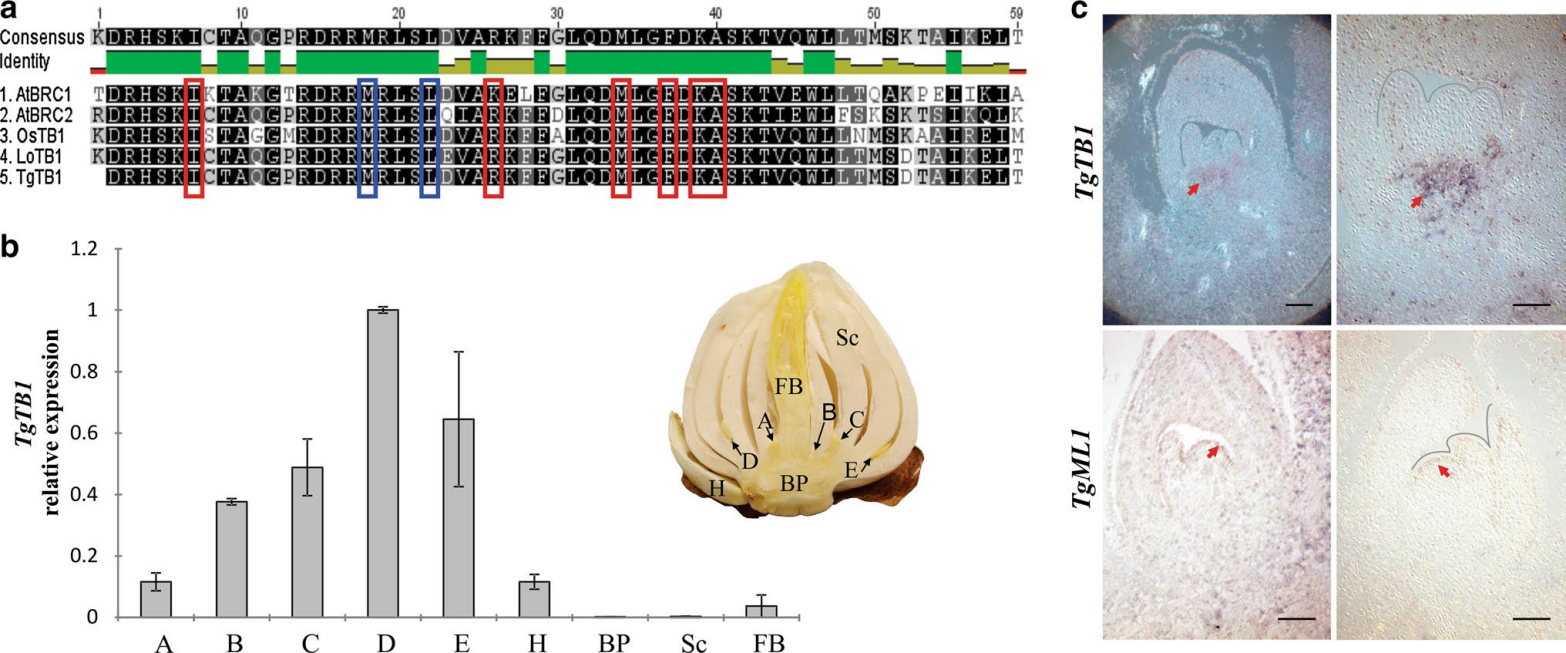
Identification of *TgTB1* sequence and its expression in different tulip bulb tissues. **a** Protein domain alignment of rice OsTB1, Arabidopsis AtBRC1 and AtBRC2 and the putative lily (LoTB1) and tulip (TgTB1) TB1/BRC1 sequences. Red boxes: conserved amino acids in CYC/TB1 proteins. Blue boxes: conserved unique amino acids in TB1/BRC1/BRC2-like proteins described by (Martín-Trillo and Cubas 2010). **b** Quantitative expression analysis of *TgTB1* in different tissues of a bulb at the end of the storage period and one day prior to planting. The expression is relative to the value of D buds. *A, B, C, D, E, H*: axillary buds located from inner to outer position in the bulb. BP: basal plate. Sc: scales. FB: floral bud. Data show the average value of three biological replicates ± SE. **c** *TgTB1* in situ hybridization in D buds from bulbs collected at the end of the storage period, one day prior to planting. Longitudinal sections probed with antisense *TgTB1* and antisense *TgML1* as a positive control (Javelle et al. 2011). The expression of *TgTB1* was observed at the base of the bud, as indicated by the arrowhead. The expression of *TgML1* was observed in the L1 layer of the meristem and scale primordia, as indicated by the arrowhead. Scale bar: 200 µm

We had previously identified several TCP domain-containing transcripts of tulip and lily, among which, one lily (*Lilium oriental*) transcript revealed a close sequence homology with Arabidopsis *BRC1* (*TCP18*) (Moreno-Pachon et al. 2016). Using primers based on the lily *TB1* (*LoTB1*) sequence, a 406-bp fragment (including the entire basic helix-loop-helix domain called TCP domain) of a potential tulip *TgTB1* transcript was isolated (Online resource OSM2). It has been reported that TB1/BRC1 proteins share two specific amino acids in the basic region of the domain, which distinguishes them from the other class II TCP transcriptions factors (Martín-Trillo and Cubas 2010). Thus, we aligned the potential tulip TgTB1, lily LoTB1, Arabidopsis AtBRC1 and rice OsTB1 protein domain sequences and found a high degree of sequence similarity, as well as the presence of the two TB1/BRC1-specific amino acid features in the isolated tulip sequence at the expected position (Fig. 2a).

*TB1/BRC1* transcripts have been detected in the meristem and leaf primordia of axillary buds of maize and in pro-vascular tissues of buds bearing flowers in Arabidopsis (Aguilar-Martínez et al. 2007; Hubbard et al. 2002). To corroborate the true *TB1/BRC1* nature of our identified *TgTB1* transcript, the expression of *TgTB1* was quantified in different organs of the bulb (axillary buds, floral bud, scales and basal plate) at the end of the storage period (prior to planting). Among all tissues tested, *TgTB1* expression was only significantly detected in the axillary buds. Moreover, active A and H buds had the lowest expression, while highest expression was found in the dormant D buds (Fig. 2b). Subsequently, in situ hybridization was carried out on D buds of bulbs at the end of the storage period, and *TgTB1* transcripts were detected in the pro-vascular tissue just below the central meristematic region (Fig. 2c).

**Fig. 3:**
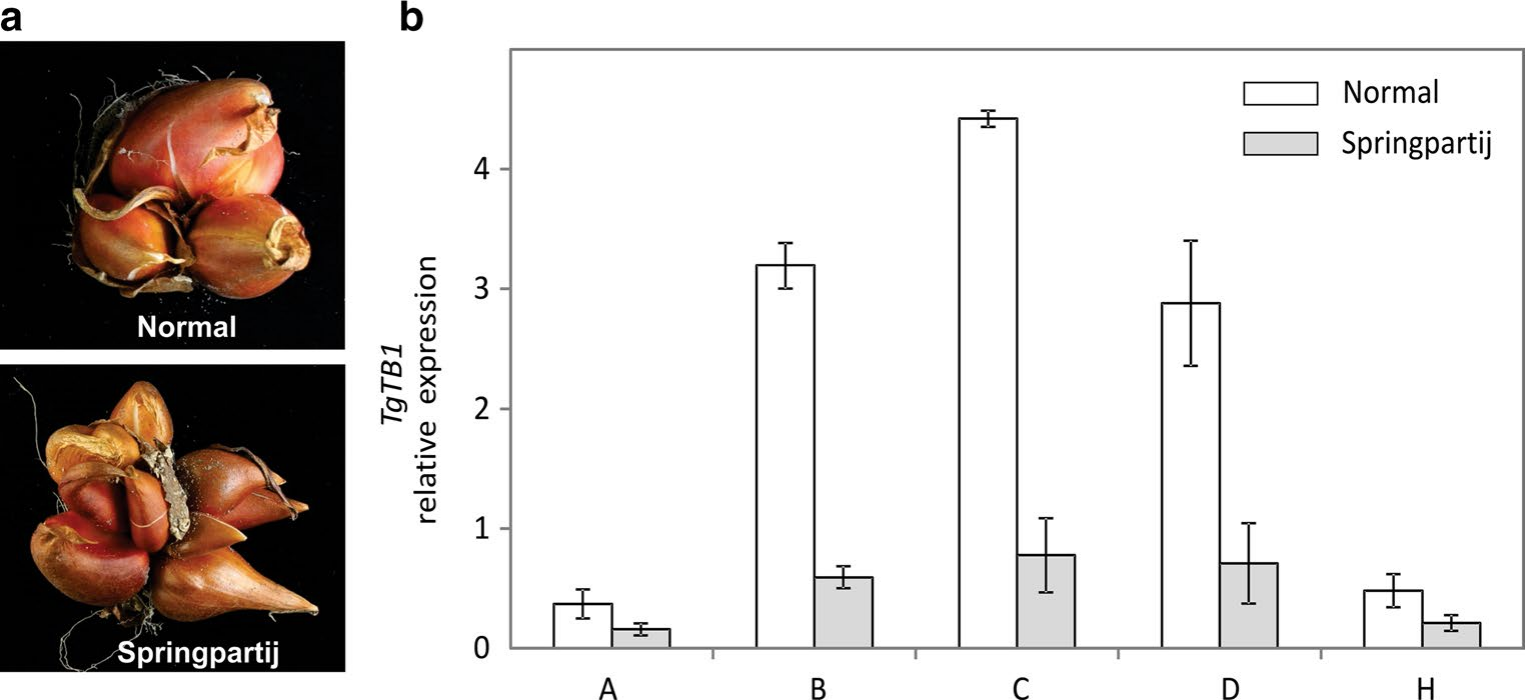
The “Springpartij” phenotype and *TgTB1* expression in axillary buds of “Springpartij” daughter bulbs. **a** Phenotype of a “Springpartij” clump of tulip daughter bulbs at harvest (down) in comparison with a normal tulip bulb clump at the same moment (up). Note that all daughter bulbs from the “Springpartij” grew out till more or less similar size. **b** Expression analysis of *TgTB1*. Normal bulbs were used as control. Clumps of daughter bulbs were lifted from the ground and stored at 20 °C for 10 weeks. The axillary buds of every daughter bulb were dissected and pooled based on their position in the bulb, as a pool of *A, B, C, D* and *H* buds. Data show the average value of three biological replicates ± SE

To investigate further the role of *TgTB1* as a marker for dormancy in axillary buds, we assessed the expression of *TgTB1* in daughter bulbs having a so-called Springpartij phenotype. “Springpartij” refers to a phenomenon experienced in some tulip bulbs where there seems to be no control in outgrowth of axillary meristems contained in the mother bulb, including the axillary buds of the axillary buds (hence, grand-daughter buds). As a consequence, many daughter bulbs of more or less the same size are formed from a single mother bulb at the end of the growing season (Fig. 3A). In general, this spontaneously occurring phenomenon cannot be reverted, and in the next growing season the phenotype will be repeated in each of the daughter bulbs. In line with the proposed function of TgTB1 in repressing axillary meristem outgrowth, we found overall low expression of *TgTB1* in ‘Springpartij’ buds (Fig. 3b) and did not observe significant differences in *TgTB1* expression among the axillary buds, as it was found in normal buds (Figs. 2b, 3b).

To study the dynamics of *TgTB1* expression in tulip axillary buds and its correlation with outgrowth, five physiological states of the growth cycle (during storage, at planting time, before anthesis, after anthesis and at lifting) were studied in A and D axillary buds. Those buds were chosen because of their contrasting behaviour in growth during the growing season (Fig. 1c), and their different *TgTB1* expression at storage (Fig. 2b).

**Fig. 4:**
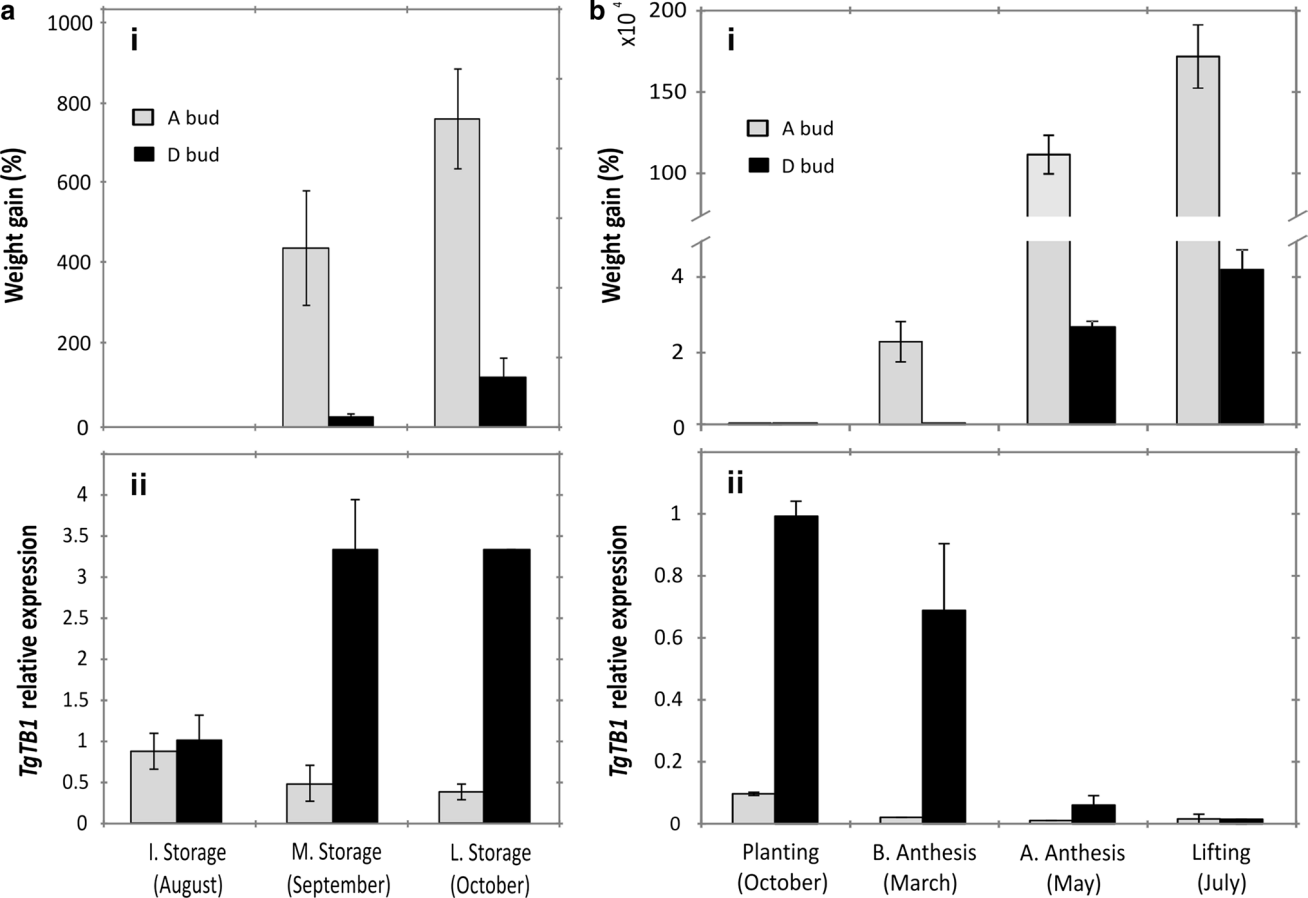
Axillary bud outgrowth in terms of weight increase and *TgTB1* expression in the innermost “*A*” and mid-located “*D*” buds during the tulip growth cycle. (*i*): Weight increase measured as weight (%) gained relative to the first time point of each bud. (*ii*): *TgTB1* expression. Data relative to the first time point of D bud. **a** Three time points were chosen to study *TgTB1* expression during storage: initial storage (I. Storage), which was the time after the formation of the *A* bud; mid storage (M. Storage), corresponding to one month after the formation of the *A* bud; and final storage (F. Storage), which was 2 months after the formation of the A bud. **b** Four time points were studied during the growing season: before planting (late October); before anthesis of the apical bud (March); after anthesis of the apical bud (May); and at the end of the growing season, also called lifting time (July). Data show the average value of three biological replicates ± SE

The results indicated that during the storage period (time previous to bulb planting), D buds did not significantly grow, while A buds experienced a linear growth increase (Fig. 4ai). Regarding the *TgTB1* relative expression during this time, there was a dramatic upregulation in D buds by the end of the storage period (late storage time), while *TgTB1* decreased in A buds (Fig. 4aii). Figure 4bii indicates that after the bulb is planted, *TgTB1* relative expression in D buds decreases also. However, the relative expression of *TgTB1* in both A and D buds becomes similar only at the moment of lifting at the end of the growing season (Fig. 4bii).

Following the idea of *TB1* expression as a marker for bud dormancy, we expected the downregulation of *TgTB1* in D buds towards the end of the growth season to result in significant bud growth. From Fig. 4bi, it can be observed that limited, although significant growth was accomplished in D buds in this last period. A possible explanation for the limited growth of D buds could be the lack of a good vascular connection. We discarded this possibility, because both A and D buds showed a main vasculature connecting them with the basal plate (Online resource OSM3).

In summary, *TgTB1* expression in the selected axillary buds was inversely correlated with their growth pattern during storage (*r* ^2^ = − 0.9) and during the growing season, however, to a lower extent (*r* ^2^ = − 0.56). When using *TgTB1* as a marker to assess dormancy, it can be said that D and not A buds enter a dormancy state during storage, and this dormancy is only broken in late spring after planting and growth during winter. However, although D buds are freed from a molecular imposed dormancy in spring, their growth is still limited and not supported.

### The role of sucrose in modulating tulip axillary bud outgrowth and *TgTB1* expression

Sugars are stored in the bulb scales as starch and translocated to the sink organs based on their demand (Ho and Rees 1976). Nevertheless, axillary buds obtain most of the carbon from the leaves of the apical bud once it sprouts in early spring (Ho and Rees 1975). Taking into account the reported role of sucrose to break bud dormancy (Barbier et al. 2015; Mason et al. 2014) and the observed correlation of *TB1/BRC1* with axillary bud growth (Aguilar-Martínez et al. 2007; Braun et al. 2012; Martín-Trillo et al. 2011; Takeda et al. 2003), we reasoned that mid-located buds might not get enough sucrose to break dormancy in early spring, probably because of competition with the already active buds (e.g. A bud).

**Fig. 5:**
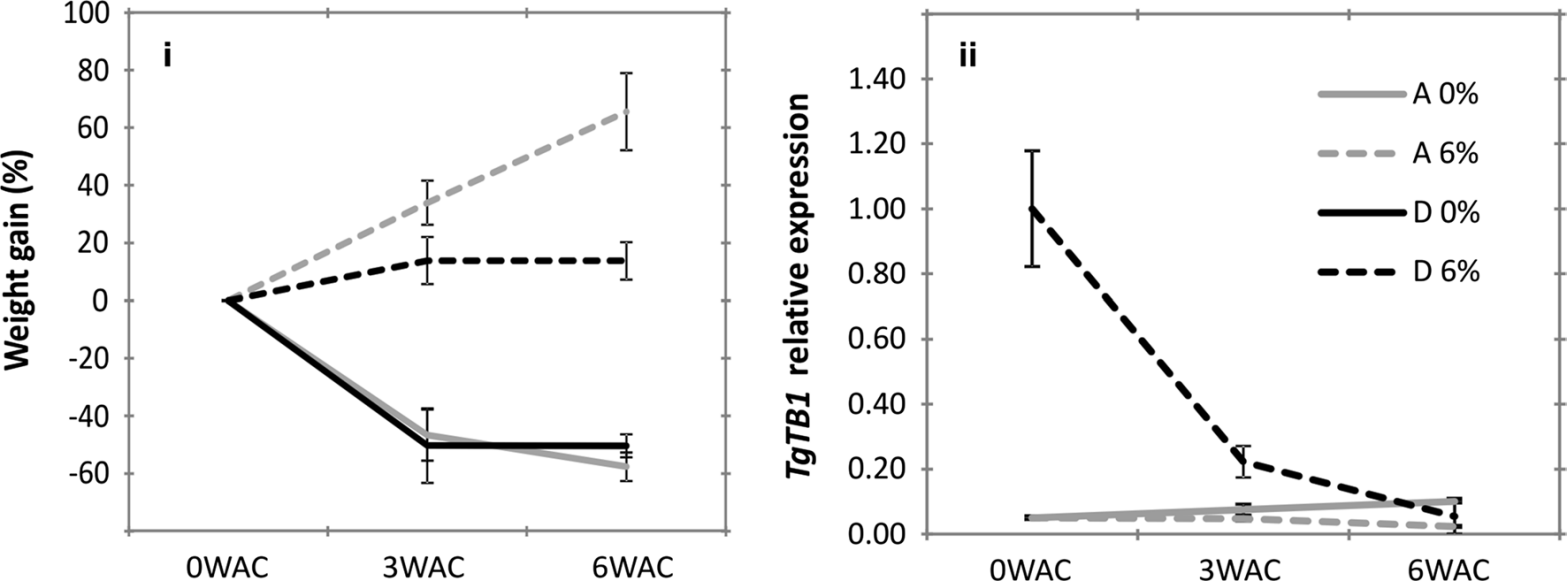
*TgTB1* expression and growth in dormant “D” and active “A” buds cultured in vitro with 0 or 6% sucrose, respectively. Bulbs were lifted and stored for 3 months at 20 °C followed by 4 °C for 3 months to mimic the end of the cold season from the natural growth cycle. (*i*) Weight increase measured as dry weight gained (%). (*ii*) *TgTB1* expression. Data shown are relative to the first time point of *D* bud. No *TgTB1* data were available for D buds without sucrose because these buds did not survive. WAC: weeks after the start of culture. Data show the average value of three biological replicates ± SE

To test whether making sucrose equally available to the buds could break dormancy and promote growth in D buds, we cultured them in vitro with or without sucrose in the medium. A buds were used as control. The buds were excised from mother bulbs stored at 20 °C for three months followed by 4 °C for another three months. These temperature pre-treatments recreated the storage and cold period of their natural growth cycle, resembling the early spring time of tulip growth cycle (Fig. 1). As expected, sucrose enhanced the growth in both buds, although to a much lesser extend in D buds (Fig. 5). Without sucrose both A and D buds lost weight and this was lethal for the majority of the smaller D buds. Hence, sucrose was fundamental for the survival of D buds, which is an indication that the buds are able to uptake the sucrose that was present in the medium. *TgTB1* expression in dormant D buds was downregulated after the sucrose treatment, as expected. However, it was intriguing that D buds did not grow significantly in the last 3 weeks of the experiment where sucrose was supplemented. As sugars as an energy source do not seem to be limiting, we speculated that other factors, such as hormones, might also play a role in controlling the growth capacity of axillary tulip buds.

### Hormonal modulators of *TgTB1*

Rameau et al. proposed a general model in which *TB1/BRC1* integrates multiple pathways that control axillary bud outgrowth (Rameau et al. 2015). In this model *TB1/BRC1* is activated by strigolactone (SL) while repressed by sucrose, cytokinin (CK) and gibberellic acid (GA). However, gibberellins can induce axillary shoot elongation instead of biomass gain in tulip [revised in (Okubo 2012)]. To investigate the role of SL and CK in the regulation of axillary bud outgrowth in tulip, we tested the combined effect of sucrose and CK (BAP) in the outgrowth of dormant D buds, since we found that sucrose is required for the survival of this type of buds. The effect of SL (GR24) was tested in the active A buds using mannitol as an osmotic control (Henry et al. 2011).

**Fig. 6:**
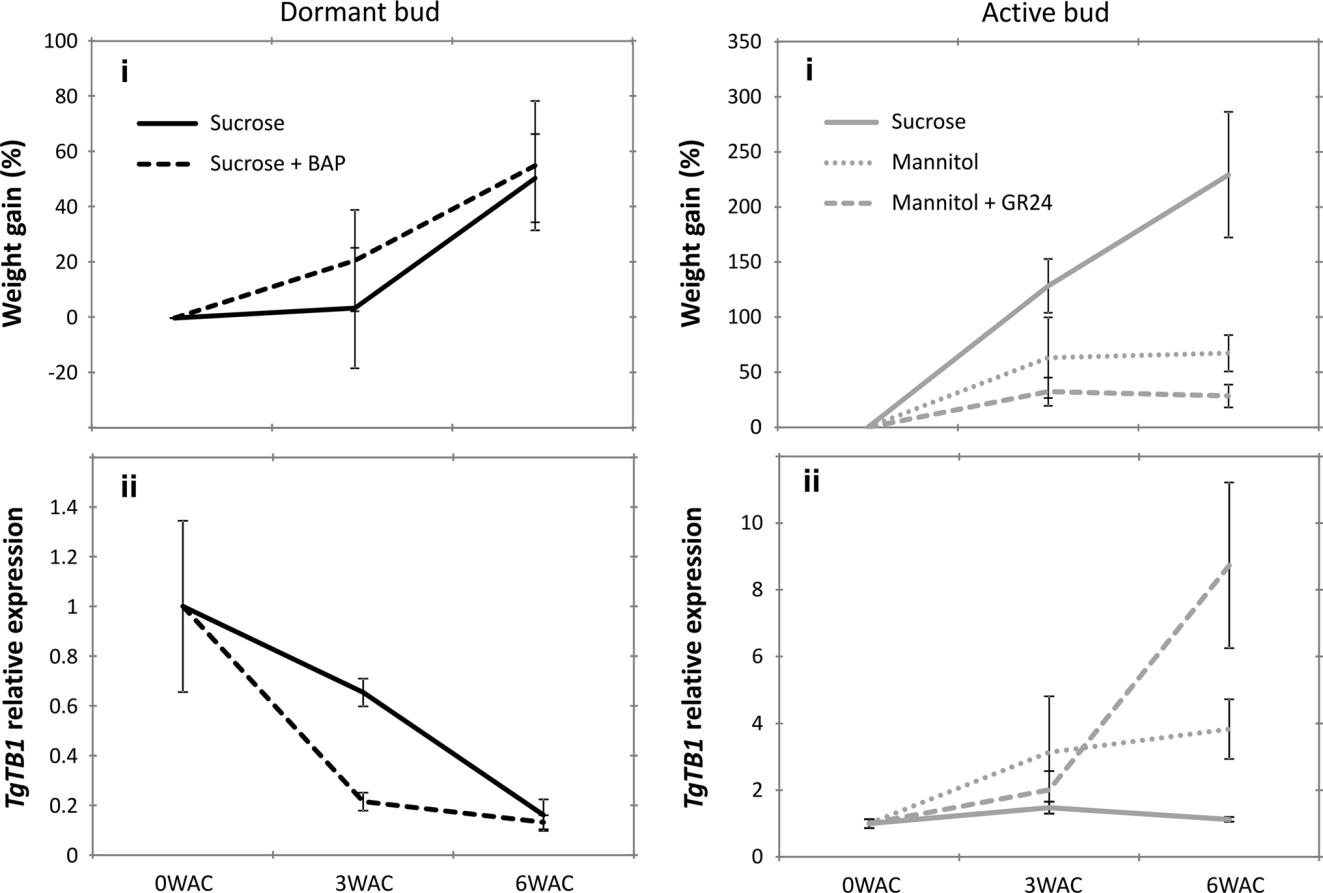
Axillary bud outgrowth and *TgTB1* expression in dormant and active buds cultured in vitro with 6% sucrose and synthetic hormones. (*i*): Weight increase measured as dry weight gained (%). (*ii*): *TgTB1* expression. Data were normalized against the first time point (0WAC). **a** Dormant *D* buds were cultured with sucrose or with the combination sucrose + 25 μM BAP (a synthetic cytokinin). **b** Active A buds were cultured with sucrose (as a positive control), mannitol (an osmotic control) or mannitol + 0.5 μM GR24 (synthetic strigolactone). Data show the average value of three biological replicates ± SE

The results showed that the combination of sucrose + BAP downregulated *TgTB1* expression faster than sucrose on its own in dormant D buds (Fig. 6aii). Nonetheless, after 6 weeks of culture, both the treatments of sugar and sugar + BAP showed the same capacity to induce bud growth (Fig. 6ai) and downregulated *TgTB1* to the same level (Fig. 6aii). With regard to A buds, they experienced an upregulation of *TgTB1* expression upon culture with mannitol and strong increase in *TgTB1* expression with the combination mannitol + GR24 (Fig. 6bii), indicating that SL can modulate *TgTB1* expression in tulip bulbs. Moreover, for both treatments, mannitol and mannitol + GR24, axillary bud outgrowth (Fig. 6bi) was negatively correlated with the level of *TgTB1* expression (Fig. 6bii).

## Discussion

Tulip bulbs can propagate via either sexual or vegetative (axillary bud outgrowth) reproduction, and both types of reproduction bring advantages and disadvantages to the survival of the species. Sexual reproduction guarantees the maintenance and broadening of the genetic pool, but the transition from seed to a flowering bulb can take up to 7 years (Minas 2007; Shahin et al. 2012). The seedling, which is at the juvenile stage, needs to form a small bulb during the first growth cycle, and thereafter, the bulb will increase its biomass in every growth cycle until it reaches the reproductive stage (Le Nard and de Hertogh 1993). Axillary bud outgrowth of a flowering-sized reproductive bulb on the other hand does not broaden the genetic variability, but it guarantees the survival of the species by shortening the time needed to produce new flowering bulbs.

The fact that in tulip not all axillary meristems arrest their growth once they have formed a bud (e.g. A bud), neither their growth results in the same size, might be a strategy of the species to ensure that at least one bud will get sufficient carbon resources to reach a flowering size. This strategy requires a complex mechanism that integrates developmental processes (when the axillary bud is initiated), molecular control of dormancy and dormancy release, sink strength of the buds and likely other or yet unidentified factors.

### Axillary bud development and molecular control of dormancy

When a flowering-sized mother bulb is planted in autumn, it has formed all its axillary buds. However, from the early spring to lifting time, grand-daughter buds (axillary bud of the axillary buds) are formed gradually from outer to inner scales (Rees 1968). It has been reported that as in many other plants, once tulip axillary (grand-daughter) buds are formed, they go into dormancy. However, the A bud escapes dormancy because it is initiated at the time the SAM transitions to the reproductive state, the moment when apical dominance is supposed to be gone (Rees 1968, 1981). We found that A and B buds are actually initiated after the SAM has transitioned to the reproductive state and that once initiated, those buds never cease to grow. We did not assess the expression of *TgTB1* at the initiation stage of each axillary bud; thus, we cannot prove that H, E, D and C buds go into a dormant state after being formed. However, based on the expression of *TgTB1* in just-formed A buds and long-time-formed D buds (at initial storage time) it seemed that only then D buds entered a dormant state. The lack of growth of those buds during storage and winter supports the idea that they are dormant.

The term dormancy in flower bulbs is controversial because it has always been assessed in terms of growth or metabolic activity (Okubo 2012). In this study, we assessed dormancy in terms of *TgTB1* expression and we found that the molecular mechanism of bud outgrowth in tulip is not that different from other plants including model species. As in pea, potato, poplar, crocus and Arabidopsis, *TgTB1* can be modulated by sucrose, cytokinin and strigolactone. Nevertheless, *TB1* downregulation in dormant buds of tulip does not always result in active growth.

### Dormancy and sink strength in tulip axillary buds

In agreement with Rees, the lack of dormancy in inner buds (A, B) seems to be caused by the lack of apical dominance once those buds are initiated. Moreover, we suggest that this lack of dormancy in inner buds makes them sink organs, even when the bulb is not photosynthetically active. Sucrose is the main product of carbon fixation during photosynthesis (Ayre 2011), and in tulip it is mobilized from the photosynthetic active leaves to the storage organs where it will convert mainly into starch (Rees 1992). Previous studies have used heavy carbon isotope labelled ^14^CO_2_ to investigate the dynamics of the source–sink relationships of the carbon balance in tulip (Ho and Rees 1975, 1976, 1977). The researchers found that in the absence of photosynthesis (storage and winter period) carbon from the bulb scales of the mother bulb sustains the slow growth of sink organs, which in order of sink strength are apical bud, root primordia and axillary buds. Later in the growth cycle, in early spring, the leaves become photosynthetic active and both leaves and mother bulb scales turn into sugar sources for the axillary buds (Ho and Rees 1975, 1976, 1977).

But if sucrose is made available to the axillary buds in early spring, why is dormancy—assessed by *TgTB1* expression level—in inner buds (D bud in this study) only broken after flower anthesis, and why is their growth not sustained by then? Our in vitro experiments showed that *TgTB1* gets downregulated when the dormant buds were cultured with sucrose. Thus, we suggest that sucrose is not being effectively remobilized to the inner buds before the flower of the mother bulb senesces. In fact, it has been proven that only upon flower senescence, the leaves become the only source of sucrose and the axillary buds become the strongest sink (Ho and Rees 1975, 1976, 1977). However, the limited growth of D buds, even when *TgTB1* expression is sharply downregulated both in vivo and in vitro, clearly indicates that *TgTB1* is surely not the only regulator of the process.

We propose that a physical factor could be responsible for such limited growth. For example, it has been reported that in perennial trees the release of bud dormancy requires the removal of callose in the plasmodesmata in order to restore the symplastic connectivity in the meristem (Rinne et al. 2001, 2011, 2016). Although we saw there is a vascular connection between the buds and the mother bulb, it is plausible to think that it might not be very functional when callose is not entirely removed from plasmodesmata in dormant buds, and therefore, less carbon resources can be remobilized into those buds. Therefore, investigating the callose deposition and removal in dormant buds might shed more light into the axillary bud outgrowth mechanism in tulip. Alternatively, the number of scales of mid-located buds might determine their growth capacity and sink strength. D buds normally contain only 2–3 scales, while A buds have on average 5 scales. Thus, it is likely that even when the right conditions are met, D buds will never grow as much as A buds because the capacity to store carbon is dependent on the number of bulb scales. In conclusion, the differences in axillary bud outgrowth capacity are regulated by the time of bud initiation in relation to the state of the SAM, the bud dormancy status and the sink strength of the bud. As in other species, TB1 in tulip is involved in integrating sugar and hormonal signals, but *TgTB1* expression on its own does not determine the outgrowth capacity in dormant buds.

## Materials and methods

### Assessing axillary bud growth in natural conditions

Bulbs of two tulip cultivars—Dynasty (Dy) and Purple Prince (PP)—of size 9–11 (cm in perimeter) were obtained from certified growers in the Netherlands and planted in early November of 2013. Three temperature data loggers (Lascar Electronics) were buried scattered in the field at the same depth as the tulip bulbs. Bulbs were sampled once just before planting and every 4 weeks during the entire growth season (from planting to lifting). Three additional sampling points were obtained during the storage period for Dynasty bulbs lifted in 2016. For every sampling time point bulbs were dug out of the ground and dissected in order to collect the axillary buds. The tissue was collected in liquid nitrogen, freeze-dried and stored at −70 °C until use for RNA isolation. The weight of the axillary buds was also measured at every sampling time point. The tissue material and information were obtained in three biological replicates, each consisting of ten bulbs.

### Assessing axillary bud growth in vitro

A and D axillary buds from certified bulbs (cooled at 4 °C for three months after the storage period, to simulate the cold winter period) were excised from the mother bulb always leaving a piece of the basal plate containing the vasculature of the bud. Excised buds were sterilized by dipping them in 70% ethanol, followed by 2% sodium hypochlorite for 20 min and three washes with sterile water for one, five and 10 min, respectively. The sterile buds were air-dried and individually cultured on solid medium (½ strength MS medium, 0.8% phytagel (w/v), pH 5.8 and 0.1% dimethyl sulphoxide (v/v)) supplemented with 6 or 0% sucrose (w/v) and/or different concentrations of 6-benzylaminopurine (BAP) (Sigma) or the synthetic SL (GR24) (provided by Binne Zwanenburg, University of Nijmegen, The Netherlands).

It has been reported that 3–6% sucrose promotes significant outgrowth of lilium bulblets cultured in vitro (Bonnier and Van Tuyl 1997; Maślanka and Bach 2014); therefore, we used 6% sucrose. For the hormone treatments, three concentrations were tested in order to find the optimal dose for the experiments: 0.5, 5 and 25 μM for GR24 and 1, 25, 50 and 100 μM for BAP. In vitro cultured buds were grown in a climate room at 24 °C in dark. The dark environment simulates the natural growth conditions of tulip bulbs underground. For each experiment three biological replicates were done (ten buds per replicate).

The weight of excised axillary buds cultured in vitro was measured at the start and after 3 and 6 weeks of culture (WAC). Initial bud fresh weight (iFW) was measured by weighing the pots with medium before and immediately after placing the buds in culture (0WAC). Final fresh weight (fFW) was measured at 3 and 6 WAC, and the bud growth was calculated as growth gain in order to correct for the differences in bud size prior to in vitro culture: [FW gain (%) = ((fFW − iFW)/iFW))*100]. After determination of weight, buds were immediately collected in liquid nitrogen, freeze-dried and stored at − 70 °C until RNA isolation.

### Identification of tulip *TgTB1*

*TB1* sequence was first identified in a *Lilium* cDNA library obtained by Illumina-based RNA sequencing (Moreno-Pachon et al. 2016) and then PCR amplified in tulip using several primers designed on the lily *TB1* sequence (Online resource OSM2). The detailed method was as follows: all lily transcripts from the library containing the TCP domain were retrieved and compared with *Oryza sativa* and *Arabidopsis thaliana* TCP proteins by the neighbour joining clustering method. The conserved features shared by the TB1/BRC1/BRC2-like proteins described by (Martín-Trillo and Cubas 2010) were used to corroborate the true lilium *TB1/BRC1* gene identity. Several primers flanking the lily *TB1* domain were used to amplify *TgTB1* in a pool of cDNA from tulip axillary buds in several stages of development. The amplified *TgTB1* fragments were sequenced, and a region of the *TB1* gene in tulip was reconstructed and named *TgTB1* (see OSM 2). The best primer combination which gave rise to a 406-bp fragment was 5′-AGGATCGCCACAGCAAGATA-3’ and 5′-TCCACCTTGTTAGCCTGACC-3′.

### Quantification of gene expression

Total RNA was isolated following the hot borate protocol (Wan and Wilkins 1994) with modifications as described by Maia et al. (2011): 60 mg of dried ground tissue was homogenized and mixed with 800 µL of extraction buffer (0.2 M Na borate decahydrate (Borax), 30 mM EGTA, 1% SDS, 1% Na deoxycholate (Na-DOC)) containing 1.6 mg DTT and 48 mg PVP40 which had been heated to 80 °C. 1 mg of proteinase K was added to this suspension and incubated for 15 min at 42 °C. After adding 64 µl of 2M KCL, the samples were incubated on ice for 30 min and subsequently centrifuged for 20 min at 12,000 g. Ice-cold 8M LiCl was added to the supernatant in a final concentration of 2M, and the tubes were incubated overnight on ice. After centrifugation for 20 min at 12,000*g* at 4 °C, the pellets were washed with 750 µl ice-cold 2 M LiCl. The samples were centrifuged for 10 min at 10,000*g* at 4 °C, and the pellets were re-suspended in 100 µl DEPC-treated water. The samples were phenol–chloroform-extracted, DNase-treated (RQ1 DNase, Promega) and further purified with RNeasy spin columns (Qiagen) following the manufacturer’s instructions. RNA quality and concentration were assessed by agarose gel electrophoresis and Expose ND (Trinean). 500 ng of total RNA was reverse-transcribed using the iScript cDNA synthesis kit (Bio-Rad). Quantitative real-time PCR (qRT-PCR) was performed using the Bio-Rad CFX96 Real-Time PCR System and SYBR Green Supermix (Bio-Rad) according to the manufacturer’s instructions. Three biological replicates, each consisting of ten buds, were used as biological replicate. qbase + software (Biogazelle) was used to determine the expression stability of five tulip candidate reference genes: *ELONGATION FACTOR 1 alpha* (*TgEF1a*)*; ACTIN* (*TgACT*)*; PROTEIN PHOSPHATASE 2* (*TgPP2*)*; ADENINE PHOSPHORIBOSYLTRANSFERASE 1* (*TgAPT1*) and *TgTIP41* (OSM 4). Quantification of *TEOSINTE BRANCHED1* (*TgTB1*) expression (primer sequences in OSM 2&4) was calculated using the ΔΔCt method, using *TgEF1α* as internal standard.

### In situ hybridization of *TgTB1* and *TgML1*

RNA in situ hybridization was performed on cross sections of “D” buds excised from bulbs during storage. The procedure was done following the protocol of Langedale (Langdale 1993). The samples were fixed overnight in 4% formaldehyde in phosphate-buffered saline (PBS), dehydrated in a an ethanol series, embedded in Paraplast Plus (Sigma-Aldrich) and sectioned 12 µm thick with a rotary microtome (Zeiss HM340E). The sections were incubated overnight with the sense and antisense probes of each gene fragment. The probes were created with in vitro transcription according to the DIG RNA Labelling Kit instructions (SP6/T7; Roche). The cDNA used for the probe transcription was a 406 base pairs *TgTB1* fragment using the primers 5′-AGGATCGCCACAGCAAGATA-3′ and 5′-TCCACCTTGTTAGCCTGACC-3′; and 614-bp *TgML1* fragment using the primers 5′-ACAACCGCTGAAAGCAACAT3′ and 5′-TCATCTGCTGGTCCCCAAAT3′. The sections were observed under light microscopy.

### Vasculature staining of axillary buds

Axillary buds attached to a piece of basal plate were embedded in 4% agarose and longitudinally sectioned by hand. The sections were stained for lignin with 2% phloroglucinol (w/v) in 95% ethanol. Subsequently, the sections were soaked in 37% (v/v) HCL. Photographs were taken within 30 min with a light dissecting microscope.

### “Springpartij” tulip material

“Springpartij” bulb clumps and normal clusters of cultivar “Prinses Catharina Amalia” were collected and stored in a dark room at 20 °C for 10 weeks. A, B, C, D and H positioned bulbs were collected, and three replicates were made. The samples were freeze-dried overnight, ground with beads and stored in − 80 °C until further use.

#### Author contribution

NMMP and RGHI conceived and designed experiments. NMMP, MCM and EH conducted most of the experiments’ data analysis. IB highly contributed to the in situ hybridizations. LS and AB assisted in in vitro culture experiments. NMMP and RGHI wrote the manuscript. HWMH revised the manuscript.

## Electronic supplementary material

Below is the link to the electronic supplementary material.

Supplementary material 1 (PDF 343 kb)
