# Supplementary material for: Role of Tulipa gesneriana TEOSINTE BRANCHED1 (TgTB1) in the control of axillary bud outgrowth in bulbs
Source: Plant Reprod. 2017 Dec 7;31(2):145–57. doi: 10.1007/s00497-017-0316-z (PMC5940712; doi:10.1007/s00497-017-0316-z)
Supplement: Supplementary file 1 — Supplementary material 1 (PDF 343 kb) [file 497_2017_316_MOESM1_ESM.pdf]

Online Resources

OSM 1

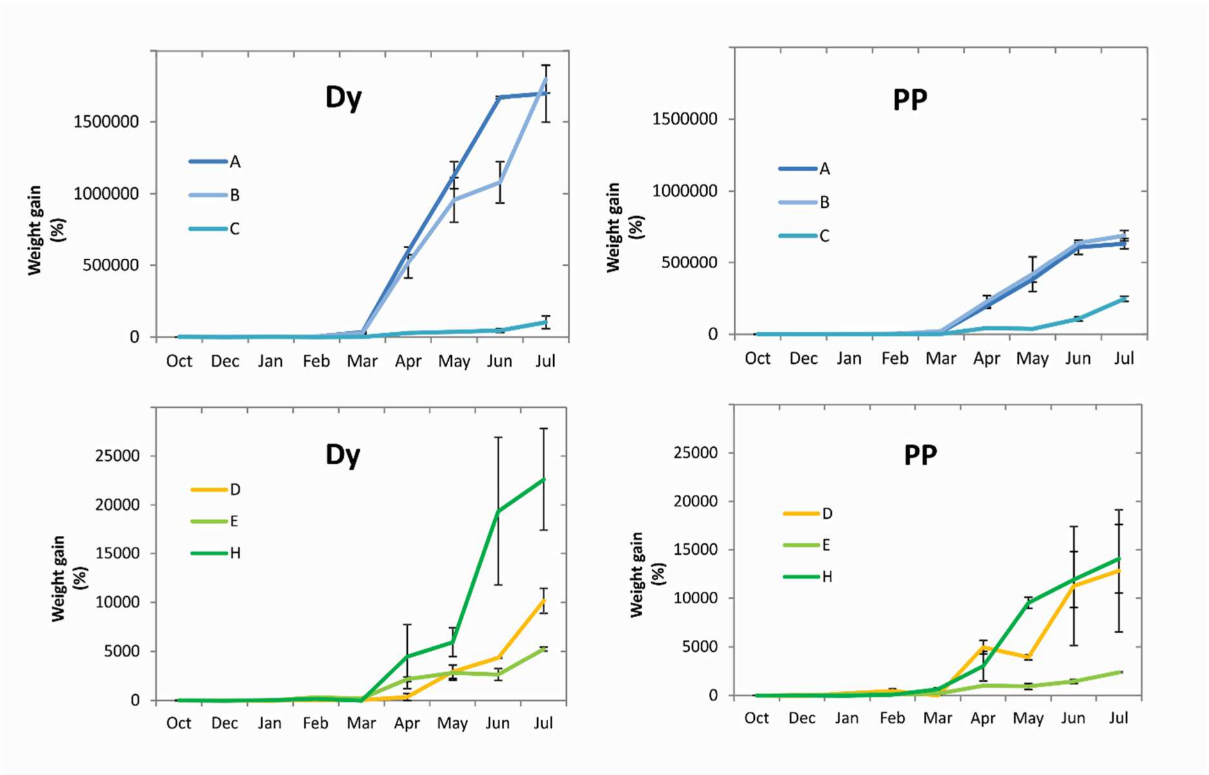

Axillary bud growth of two tulip cultivars at several time points of the growing season. Weight increase was assessed as fresh weight gain in percentage (%). Dy: cultivar Dynasty PP: cultivar Purple Prince. Note the different Y-axis scale for A-C and D-H buds, respectively.

OSM 2

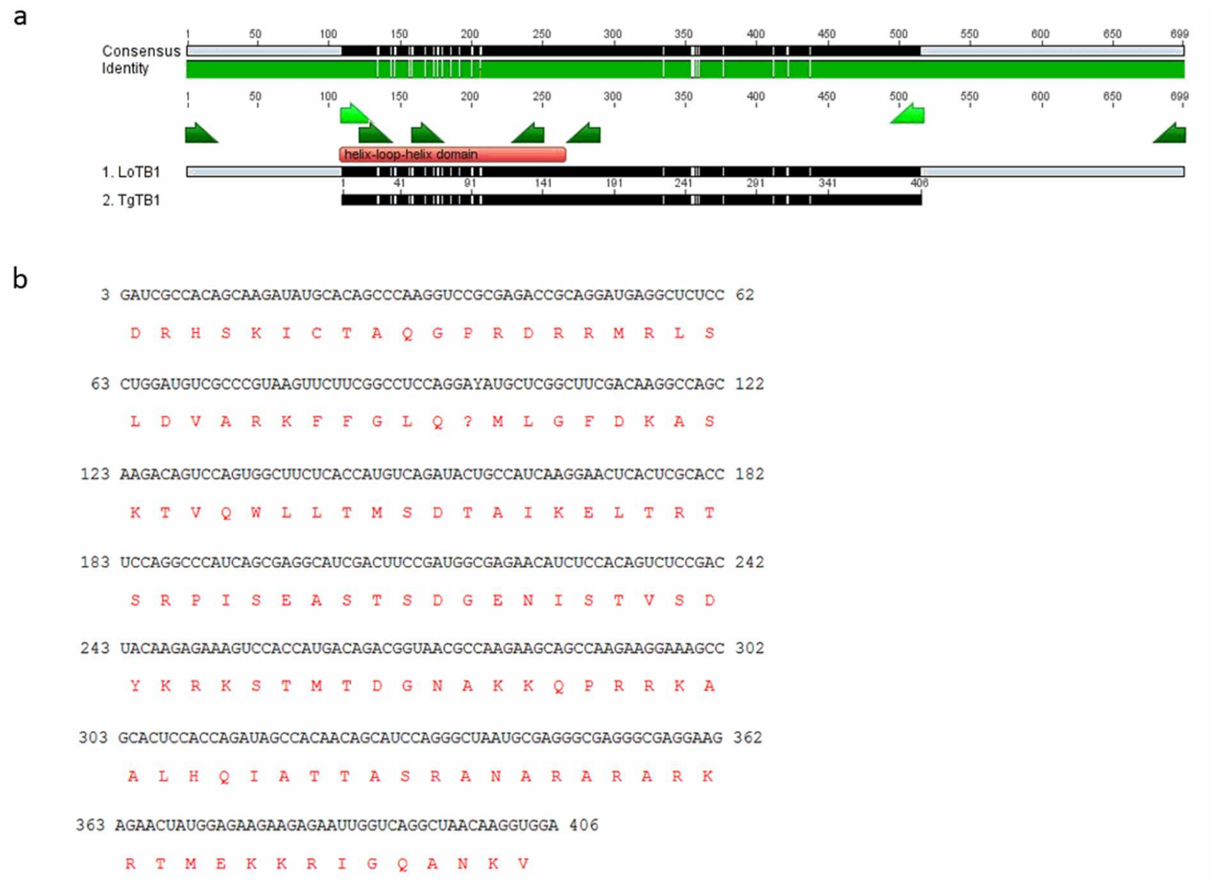

Identification of *Tulipa gesneriana TB1* (*TgTB1*). (a) Several primers (green arrows) were designed flanking the TCP domain (in red) of *Lilium TB1* (*LoTB1*). The different primer combinations were used for PCR amplification on a pool of cDNA from tulip axillary buds in several stages of development. The different obtained amplicons were sequenced and aligned to reconstruct *TgTB1*. (b) Based on the constructed putative *TgTB1* transcript fragment, two primers were designed (Light green arrows in (a); representing the primers 5′-AGGATCGCCACAGCAAGATA-3’ and 5′-TCCACCTTGTTAGCCTGACC-3’), which gave rise to the 406 base-paired (bp) fragment of *TgTB1*. Both the RNA sequence and predicted protein sequence are shown.

OSM 3

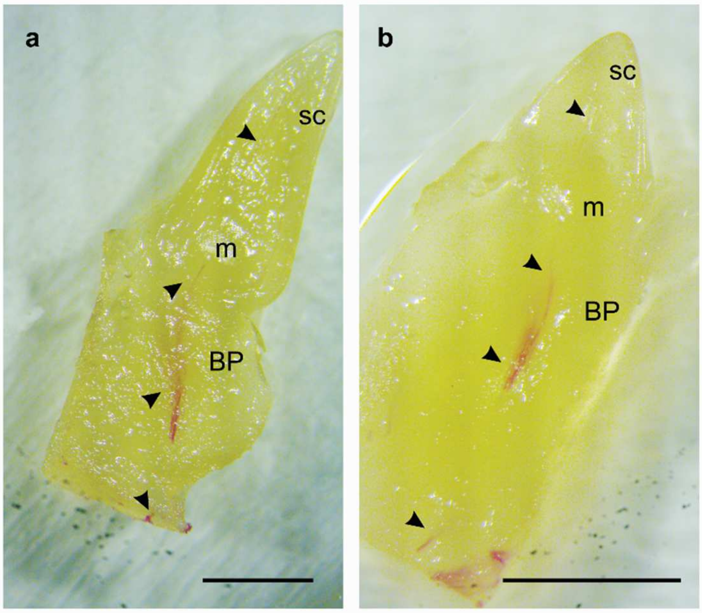

Vasculature of “D” and “A” buds. Buds (cultivar Dynasty) were excised from the mother bulb during the storage period and stained with floroglucinol-HCl, which stains lignin. (a) Axillary bud “D”. (b) Axillary bud “A”. sc: scale, m: meristem, BP: basal plate. Arrow heads indicate the position of the main vasculature, which stained red. Scale bar: 3 mm.

OSM 4

Primers used for qPCR analysis.

Gene	TAIR ID	Primer sequence
ELONGATION FACTOR 1α (TgEF1α)	At5g60390	Fw: 5' TGA GAA GGA GGC TGC TGA AA 3'<br>Rv: 5' TCA CGA TGA CCA GGA GCA TC 3'
ACTIN (TgACT)	At2g37620	Fw: 5' AGC AAC TGG GAT GAC ATG GA 3'<br>Rv: 5' GGA CAG CCT GAA TTG CAA CA 3'
ADENINE PHOSPHORIBOSYLTRANSFERASE 1 (TgAPT1)	At1g27450	Fw: 5' ATC GCT ACT GGA GGG ACC TT 3'<br>Rv: 5' AAT TGG AAC TGC GAC AAT CC 3'
TgTIP41	At4g34270	Fw: 5' GAA GCC AAA AAC GGA GAA GA 3'<br>Rv: 5' CCT GGG AAG AAT GTG ACG AT 3'
PROTEIN PHOSPHATASE 2A (TgPP2A)	At1g13320	Fw: 5' TGG CGA GTG GTT TAC TGC TA 3'<br>Rv: 5' CCG TCT TCA AAT GGT TTG GT 3'
TEOSINTE BRANCHED 1 (TgTB1)	At3g18550	Fw: 5' ATG AGG CTC TCC CTG GAT GT 3'<br>Rv: 5' ACA TGG TGA GAA GCC ACT GG 3'
